# A Comparative Analysis of Influenza Vaccination Programs

**DOI:** 10.1371/journal.pmed.0030387

**Published:** 2006-10-03

**Authors:** Shweta Bansal, Babak Pourbohloul, Lauren Ancel Meyers

**Affiliations:** 1 Computational and Applied Mathematics, University of Texas Austin, Austin, Texas, United States of America; 2 UBC Centre for Disease Control, University of British Columbia, Vancouver, British Columbia, Canada; 3 Section of Integrative Biology and Institute for Cellular and Molecular Biology, University of Texas Austin, Austin, Texas, United States of America; 4 External Faculty, Santa Fe Institute, Santa Fe, New Mexico, United States of America; Pennsylvania State University, United States of America

## Abstract

**Background:**

The threat of avian influenza and the 2004–2005 influenza vaccine supply shortage in the United States have sparked a debate about optimal vaccination strategies to reduce the burden of morbidity and mortality caused by the influenza virus.

**Methods and Findings:**

We present a comparative analysis of two classes of suggested vaccination strategies: mortality-based strategies that target high-risk populations and morbidity-based strategies that target high-prevalence populations. Applying the methods of contact network epidemiology to a model of disease transmission in a large urban population, we assume that vaccine supplies are limited and then evaluate the efficacy of these strategies across a wide range of viral transmission rates and for two different age-specific mortality distributions.

We find that the optimal strategy depends critically on the viral transmission level (reproductive rate) of the virus: morbidity-based strategies outperform mortality-based strategies for moderately transmissible strains, while the reverse is true for highly transmissible strains. These results hold for a range of mortality rates reported for prior influenza epidemics and pandemics. Furthermore, we show that vaccination delays and multiple introductions of disease into the community have a more detrimental impact on morbidity-based strategies than mortality-based strategies.

**Conclusions:**

If public health officials have reasonable estimates of the viral transmission rate and the frequency of new introductions into the community prior to an outbreak, then these methods can guide the design of optimal vaccination priorities. When such information is unreliable or not available, as is often the case, this study recommends mortality-based vaccination priorities.

## Abstract 2

A comparative analysis of two classes of suggested vaccination strategies, mortality-based strategies that target high-risk populations and morbidity-based strategies that target high-prevalence populations.

## Editors' Summary

**Background.:**

Influenza—a viral infection of the nose, throat, and airways that is transmitted in airborne droplets released by coughing or sneezing—is a serious public health threat. Most people recover quickly from influenza, but some individuals, especially infants, old people, and individuals with chronic health problems, can develop pneumonia and die. In the US, seasonal outbreaks (epidemics) of flu cause an estimated 36,000 excess deaths annually. And now there are fears that avian influenza might start a human pandemic—a global epidemic that could kill millions. Seasonal outbreaks of influenza occur because flu viruses continually change the viral proteins (antigens) to which the immune system responds. “Antigenic drift”—small changes in these proteins—means that an immune system response that combats flu one year may not provide complete protection the next winter. “Antigenic shift”—large antigen changes—can cause pandemics because communities have no immunity to the changed virus. Annual vaccination with vaccines based on the currently circulating viruses controls seasonal flu epidemics; to control a pandemic, vaccines based on the antigenically altered virus would have to be quickly developed.

**Why Was This Study Done?:**

Most countries target vaccination efforts towards the people most at risk of dying from influenza, and to health-care workers who are likely come into contact with flu patients. But is this the best way to reduce the burden of illness (morbidity) and death (mortality) caused by influenza, particularly at the start of a pandemic, when vaccine would be limited? Old people and infants are much less likely to catch and spread influenza than school children, students, and employed adults, so could vaccination of these sections of the population—instead of those most at risk of death—be the best way to contain influenza outbreaks? In this study, the researchers used an analytical method called “contact network epidemiology” to compare two types of vaccination strategies: the currently favored mortality-based strategy, which targets high-risk individuals, and a morbidity-based strategy, which targets those segments of the community in which most influenza cases occur.

**What Did the Researchers Do and Find?:**

Most models of disease transmission assume that each member of a community is equally likely to infect every other member. But a baby is unlikely to transmit flu to, for example, an unrelated, housebound elderly person. Contact network epidemiology takes the likely relationships between people into account when modeling disease transmission. Using information from Vancouver, British Columbia, Canada, on household size, age distribution, and occupations, and other factors such as school sizes, the researchers built a model population of a quarter of a million interconnected people. They then investigated how different vaccination strategies controlled the spread of influenza in this population. The optimal strategy depended on the level of viral transmissibility—the likelihood that an infectious person transmits influenza to a susceptible individual with whom he or she has contact. For moderately transmissible flu viruses, a morbidity-based vaccination strategy, in which the people most likely to catch the flu are vaccinated, was more effective at containing seasonal and pandemic outbreaks than a mortality-based strategy, in which the people most likely to die if they caught the flu are vaccinated. For highly transmissible strains, this situation was reversed. The level of transmissibility at which this reversal occurred depended on several factors, including whether vaccination was delayed and how many times influenza was introduced into the community.

**What Do These Findings Mean?:**

The researchers tested their models by checking that they could replicate real influenza epidemics and pandemics, but, as with all mathematical models, they included many assumptions about influenza in their calculations, which may affect their results. Also, because the contact network used data from Vancouver, their results might not be applicable to other cities, or to nonurban areas. Nevertheless, their findings have important public health implications. When there are reasonable estimates of the viral transmission rate, and it is known how often influenza is being introduced into a community, contact network models could help public health officials choose between morbidity- and mortality-based vaccination strategies. When the viral transmission rate is unreliable or unavailable (for example, at the start of a pandemic), the best policy would be the currently preferred strategy of mortality-based vaccination. More generally, the use of contact network models should improve estimates of how infectious diseases spread through populations and indicate the best ways to control human epidemics and pandemics.

**Additional Information.:**

Please access these Web sites via the online version of this summary at http://dx.doi.org/10.1371/journal.pmed.0030387.

- US Centers for Disease Control and Prevention information about influenza for patients and professionals, including key facts on vaccination
- US National Institute of Allergy and Infectious Diseases feature on seasonal, avian, and pandemic influenza
- World Health Organization fact sheet on influenza, with links to information on vaccination
- UK Health Protection Agency information on seasonal, avian, and pandemic influenza
- MedlinePlus entry on influenza

## Introduction

In response to the 2004–2005 influenza vaccine shortage, the United States Centers for Disease Control and Prevention (CDC) restricted vaccines to those most at risk for hospitalization and death — healthy infants, elderly individuals, and individuals with chronic illnesses. This strategy may be limited by the failure of vaccines to yield adequate protection for high-risk individuals [1,2] and the lesser roles played by infants and the elderly in disease transmission—they typically do not introduce influenza into households or other social groups.

Influenza outbreaks are believed to hinge, instead, on transmission by healthy school children [3–6], college students, and employed adults who have many daily contacts and are highly mobile [7]. Thus, epidemiologists have suggested an alternative approach: vaccinate school-age children to slow the spread of disease and thereby indirectly decrease mortality [8,9]. Several studies support this strategy. Monto et al. immunized school children in Tecumseh, Michigan, with inactivated influenza vaccine in 1968 and found lower total morbidity than in a matching community during a wave of influenza A (H3N2) [10]. Reichart et al. argue that mandatory influenza vaccination of school children in Japan from 1962 to 1987 reduced incidence and mortality among the elderly [11]. Recently, Longini et al. used mathematical models to show that, under certain assumptions, vaccinating 80% of all school-age children is almost as effective as vaccinating 80% of the entire population [8]. School-based vaccination programs have the additional benefits of high coverage, high efficacy, and minimal side effects [12].

In a similar spirit, others have suggested contact-based priorities that target individuals with the highest numbers of potentially disease-causing contacts [13–15]. This assumes that vulnerability is directly proportional to the number of contacts, and that removing the most vulnerable individuals from the transmission chain will maximally decrease disease spread. Identifying high-contact individuals in a community, however, may be difficult in practice.

Here we apply tools from contact network epidemiology [16–19] to evaluate vaccination strategies for a spectrum of influenza strains when vaccine supplies are limited. We use a realistic model of contact patterns in an urban setting to compare mortality-based strategies that target high-risk individuals to morbidity-based strategies that target demographics with high attack rates. We assess the efficacy of these measures for two substantially different virulence patterns, one based on mortality estimates from annual influenza epidemics and the other based on mortality estimates from the 1918 influenza pandemic. In addition, we consider the impact of vaccination delay and multiple imported cases on the relative effectiveness of the vaccination strategies.

## Methods

### Population Model

We built a contact network model that captures the interactions that underlie respiratory disease transmission within a city. The model is based on demographic information for Vancouver, British Columbia, Canada. In the model, each person is represented as a vertex, and interactions between people are represented as edges between appropriate vertices. Each person is assigned an age based on Vancouver census data, and age-appropriate activities (school, work, hospital, etc.). Interactions among individuals reflect household size, employment, school, and hospital data for Vancouver. The model population includes ~257,000 individuals. For further details and sensitivity analysis, see Protocol S1 and Figures S1 and S2.

**Figure 1:**
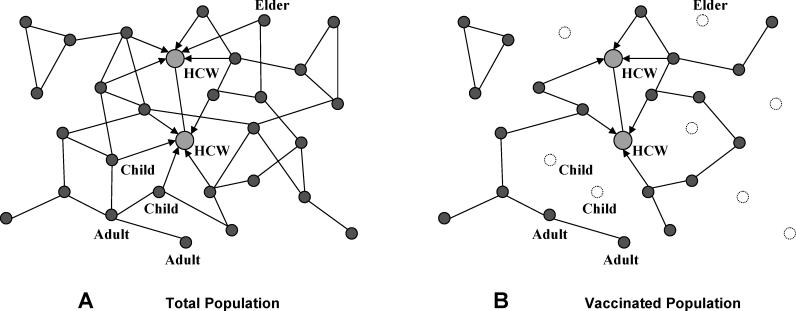
Network Model (A) A schematic of a network model for an urban population. Each individual is a vertex in the network, and edges represent potentially disease-causing contacts between individuals. Directed edges (with arrows) represent transmission occurring in only one direction. (B) We model vaccination in a population by removing nodes from the population network, and the edges that are attached to them. HCW, health-care worker.

 Our contact network model contains undirected edges that reflect the possibility of disease transmission in either direction between two individuals, and directed edges that indicate the possibility of disease transmission from one person to another, but not the reverse (see Figure 1). Directed edges model the possibility of transmission from an infected member of the general public to health-care workers during hospital visits. In a typical epidemic, most individuals infected with influenza do not seek hospital care. We assume that only high-risk groups (infants and elderly) visit hospitals upon infection and thus have opportunities to infect the health-care workers who treat them [20]. We also consider a more extreme scenario in which almost all infected individuals are at risk for serious complications and thus seek medical care upon infection.

### Influenza Mortality

Mortality rates differ both across demographic groups and among strains of influenza (see Table 1 and Protocol S1), and thus the optimal vaccination priorities are likely to depend on the virulence of the circulating strains. We consider two substantially different mortality models. The first assumes age-specific mortality rates typical of interpandemic outbreaks of flu, which are based on national viral surveillance data reported for 1977–1999 [21]. The rate is highest for the elderly, followed by infants, who are most at risk for death caused directly by influenza or pneumonia or by primary respiratory or circulatory complications. The second model, which was intentionally chosen for contrast, assumes mortality rates to be as estimated for the 1918 flu pandemic. These are high for healthy young adults aged 20–40 y and children under 5 y and low for older children and the elderly [22] (Table 1). There are, however, conflicting estimates for the elderly [23,24]. We use a low estimate to achieve the greatest departure from the interpandemic model, and thus to ascertain the sensitivity of our results to assumptions about influenza mortality. Henceforth, we refer to these two models as interpandemic and pandemic, respectively. We consider other reported mortality rates in Protocol S1 and Figure S3.

**Table 1:**
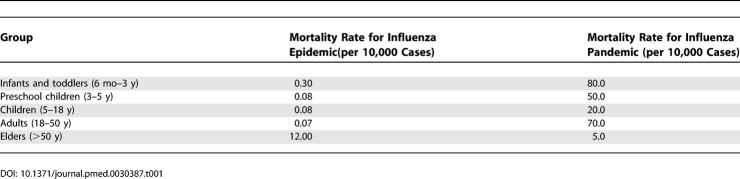
The Age-Specific Mortality Distributions for Typical Annual Influenza Epidemics and an Example Influenza Pandemic

### Vaccine Priorities

We modeled targeted pre-season vaccination with single doses of inactivated influenza vaccine by removing select individuals (vertices) and all their contacts (edges) from the network before predicting the spread of influenza (see Figure 1). The fraction of the vaccinated population that becomes fully protected is based on demographic-specific vaccine efficacy estimates (Table 2). For a vaccine of efficacy *E* and coverage *C* for a particular group, we remove a fraction *C·E* of individuals from that group. This assumes that this fraction is fully protected (100% effectiveness) while the remaining fraction *C·*(1 − *E*) of vaccinated individuals are not protected at all. In reality, most of the vaccinated individuals will enjoy some partial protection. We have tested our method with simulations to confirm that it provides a reasonable model for partial efficacy (see Protocol S1 and Figure S5).

**Table 2:**
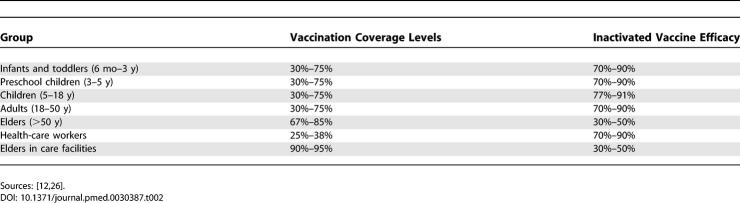
Historical Influenza Vaccination Coverage Levels and Inactivated Vaccine Efficacy Levels Used in This Study

**Figure 2:**
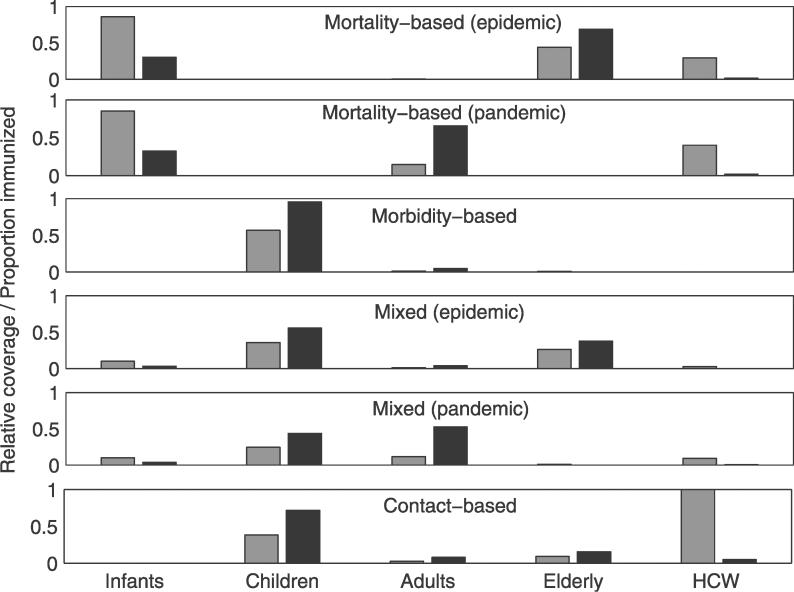
Vaccination Strategies The demographic distribution of vaccines according to each of the strategies: the black bars reflect the fraction of available vaccines given to each age group (and thus will always sum to one). The gray bars reflect the proportion of each demographic that is effectively immunized, and thus take into account the size of the demographic and the demographic-specific vaccine efficacy. HCW, health-care worker.

We evaluate four strategies (Figure 2): (1) a mortality-based strategy that, like the recent CDC strategy, targets demographics that are most vulnerable to health complications or death (infants, the elderly, and health-care workers for interpandemic flu; and infants, adults, and health-care workers for pandemic flu); (2) a morbidity-based strategy, similar to the priorities suggested by Longini et al [8] and Monto et al.[10], that targets school-aged children and school staff, and thereby aims to reduce mortality through herd protection [25]; (3) a mixed strategy that targets demographics with high attack rates (children) and high mortality rates (infants and the elderly for interpandemic flu; infants and adults for pandemic flu), similar to that suggested by Longini and Halloran [9]; and (4) a contact-based strategy that removes a fraction of the most connected individuals.

We modeled the mortality-based strategy by removing infants, the elderly, and health-care workers from the network based on reported maximum coverage and efficacy levels for these demographics [12,26] (Table 2). This yielded 13% coverage of the total population (Table 3). We then implemented the remaining strategies to match this overall coverage level. (We consider the sensitivity of our results to the coverage level in Protocol S1 and Figure S4.) Targeted groups were removed in proportion to demographic-specific vaccine coverage levels reported in the 2002 National Health Interview Survey by the CDC [26], and the vaccine efficacy levels were based on age-specific rates reported for inactivated influenza vaccine [12].

**Table 3:**
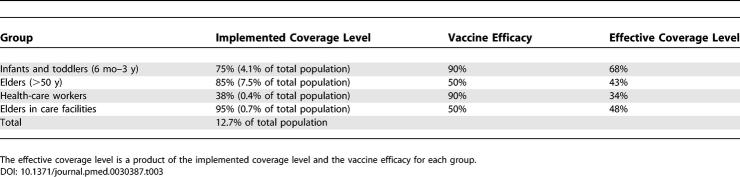
Vaccination Coverage and Efficacy Levels Assumed for the Mortality-Based Vaccination Strategy

### Epidemiological Analysis

We define the transmissibility of a disease, *T*, as the average probability that an infectious individual will transmit the disease to a susceptible individual with whom he or she has contact. This term summarizes important features of disease propagation including the contact rates among individuals, the duration of the infectious period, and the per contact probability of transmission. This per contact probability of transmission, in turn, summarizes the susceptibility (immune response) and the infectiousness (viral shedding) of individuals. Our analysis allows for variation in transmission rates from one individual to the next, but it assumes that these rates vary randomly with respect to the underlying contact patterns. There is evidence, however, that transmission rates may vary systematically among demographics, and, in particular, may be highest for children [27]. In Protocol S1 and Figure S6, we consider modified models that explicitly capture such demographic-specific variation in transmission rates and show that this additional complexity does not alter the results reported below.

*T* is linearly related to the key epidemiological parameter *R*_0_ *.* In particular, *R*_0_ is equal to *T·*κ, where κ is a measure of the connectivity within the population (network) [19,28]. Intuitively, *R*_0_ is largest for highly contagious pathogens (represented by a high *T*) spreading through densely connected populations (represented by a high κ). *R*_0_ = 1 corresponds to a critical transmissibility value *T*_c_, above which a population is vulnerable to large-scale epidemics and below which only small outbreaks occur [28].

We used methods based on contact network epidemiology [16–19] to predict the fate of an influenza outbreak as a function of the average transmissibility *T* of the strain. For any contact network, one can mathematically predict the epidemic threshold (*T*_c_), the average size of a small outbreak *(s),* the average size (*S*_e_) and probability (*P*_e_) of a large-scale epidemic, and demographic-specific attack rates for an epidemic, should one occur. Mortality is predicted by multiplying the expected number of infections for a given group by the age-specific mortality rate assumed for that group. (See Protocol S1 for additional details.)

**Figure 3:**
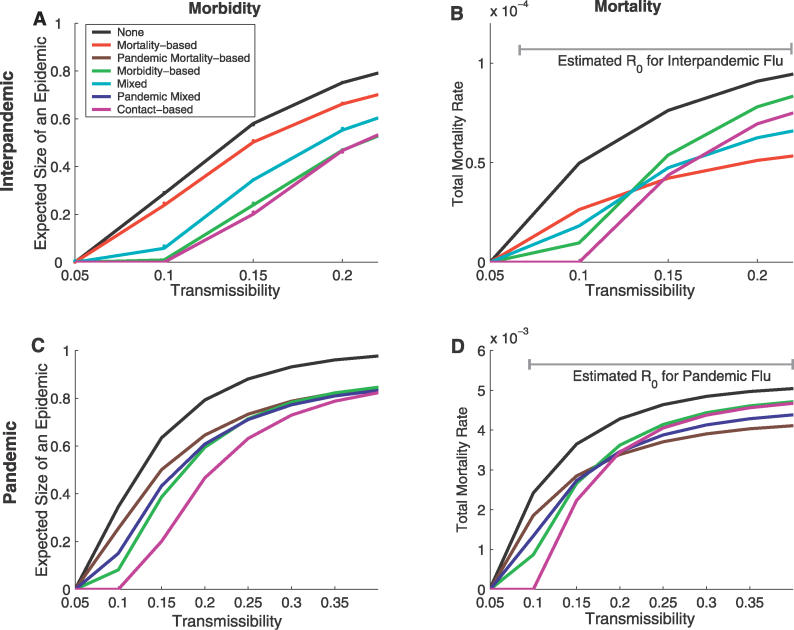
Morbidity and Mortality for Influenza Epidemics and Pandemics Expected (A) attack rate and (B) mortality rate as a function of *T* for annual influenza epidemics. Expected (C) attack rate and (D) mortality rate as a function of *T* for an influenza pandemic. The dots in (A) show simulation results for comparison. Estimates of *R*_0_ for interpandemic and pandemic flu are shown as gray lines in (B) and (D), respectively.

To verify these mathematical predictions, we performed numerical simulations of disease spread assuming a simple susceptible–infectious–recovered (SIR) model. Beginning with a susceptible network and a single infected case, we iteratively take each currently infected vertex, infect each of its susceptible contacts with probability *T,* and then change the status of the original vertex to “recovered.” These simulations are generally consistent with the mathematical calculations (Figure 3), and thus we primarily report the analytical results.

Immunity from prior outbreaks is an important aspect of interpandemic influenza transmission. There are two alternative approaches to modeling residual immunity. One is to remove individuals with naturally acquired immunity from the network, as we have done for vaccination. The other is to assume that the distribution of transmission probabilities reflects pre-existing immunity. If there is widespread partial immunity, then there will be large numbers of edges along which transmission is very unlikely, leading to a lower average transmissibility across the population. Here we have not removed individuals with naturally acquired immunity from the population, but instead assume that the transmissibility values are averaged over all edges in the network, including those leading to or from such individuals.

### Model Validation

**Figure 4:**
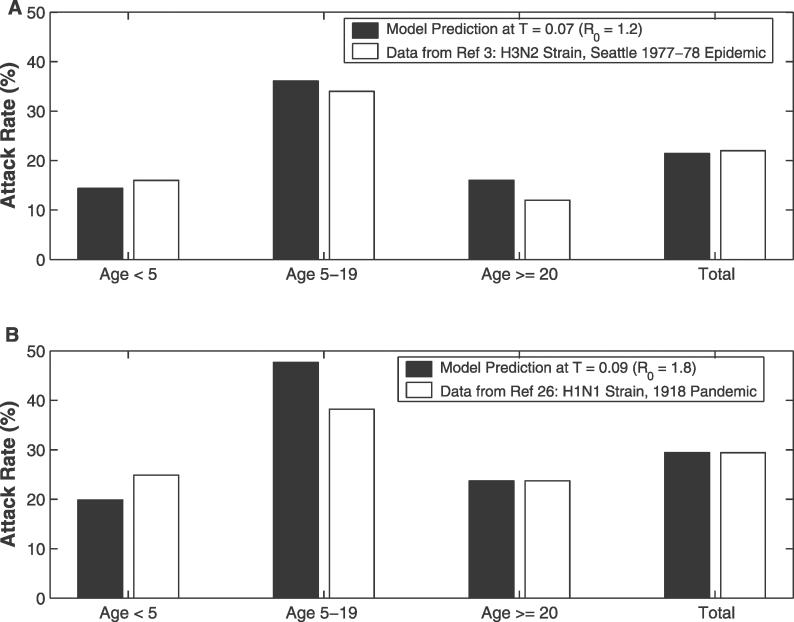
Model Validation Comparison of predicted to observed age-specific attack rates for (A) the 1977–1978 influenza season and (B) the 1918 influenza pandemic.

We compared the age-specific attack rates predicted by our models to those reported for both interpandemic flu and the 1918 pandemic (Figure 4). First, we considered data from the interpandemic outbreak of 1977–1978 reported by Longini et al. [3]. They reported age-specific attack rates from a household study of 159 families in Seattle, Washington, United States, in which infection was determined through hemagglutination-inhibition assays. We do not know the exact age-specific influenza vaccination coverage rates during this period. We assumed that the population was protected according the current CDC strategy (the mortality-based strategy), and then used our model to predict demographic-specific attack rates. We estimated the average transmissibility of the disease by solving for the value of *T* that produces the observed total attack rate (*T* = 0.07, or *R*_0_ = 1.2). Thus, the total attack rate was constrained to match perfectly the observed total attack rate, while the demographic-specific attack rates were free to vary. The predictions of the model are consistent with the observed epidemiology (Figure 4A). We note, however, that the reliability of this comparison is limited by the small sample size of the Seattle study and the lack of information about vaccine coverage and efficacy during that period.

Second, we made a similar comparison using age-specific attack rate data for the 1918 pandemic that were collected and reported by Frost in 1920 [29]. The data are based on a survey of approximately 146,000 people (representing a cross-section of the US population, which at the time numbered 103 million). Infection rates for influenza were based on self-reported responses by study participants. There was no vaccination available for influenza at the time in the US, and thus we made epidemiological predictions assuming no vaccination. Again, we began by solving for an average transmissibility that produces the observed total attack rate and found *T* = 0.09 (or *R*_0_ = 1.8). As a consistency check, this estimate agrees very closely with the recently revised estimate for the pandemic influenza reproductive rate [30], based on US and UK 1918 pandemic mortality data. Assuming this average transmissibility, we predicted demographic-specific attack rates and found that they matched the observed patterns reasonably well (Figure 4B).

## Results/Discussion

### Direct versus Indirect Intervention Methods

For interpandemic influenza, morbidity-based and contact-based strategies appear to offer significant indirect protection of unvaccinated individuals who would otherwise become infected via transmission chains that have now been severed by vaccination. Indeed, for all strains, these two strategies are predicted to yield the lowest attack rates (Figure 3A). If the primary objective is to reduce morbidity from influenza, then the morbidity-based and contact-based strategies are always preferred, although their advantage decreases as disease transmissibility *(T)* increases.

**Figure 5:**
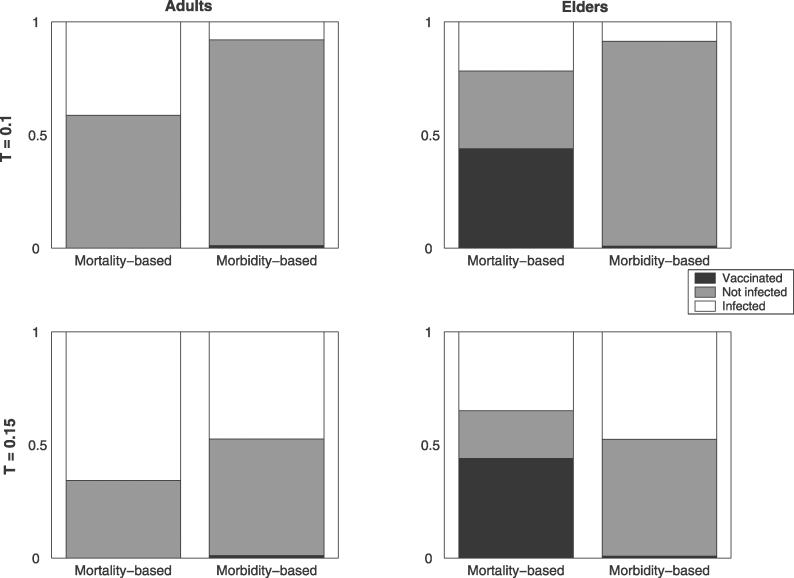
Direct versus Indirect Intervention The figure shows the proportions of the adult and elderly populations that are infected, not infected (neither vaccinated nor infected), and vaccinated for two different values of *T* for the mortality-based strategy versus the morbidity-based strategy.

One might argue that the primary objective of intervention should be to reduce mortality rather than morbidity. The CDC's recent vaccine priorities seem to be based on this objective [12]. In terms of mortality, there is a specific transmissibility value below which the morbidity-based and contact-based strategies are superior and above which the mortality-based strategies are superior (Figure 3B). To clarify this transition (which occurs for our network at *T* = 0.13), we show in Figure 5 the proportions of the adult and elderly subpopulations that are infected, vaccinated, and uninfected for the two strategies at two values of *T*. The uninfected class is made up of individuals that have neither been vaccinated nor get infected. Some of these individuals would not be infected in any case, and the rest are those that would be infected without a vaccination program but are now protected by the effects of herd immunity. Below the transition point (for instance, at *T* = 0.1), the elderly are protected more by the indirect effects of the morbidity-based strategy than by the direct effects of the mortality-based strategy. Above the transition point (for instance, at *T* = 0.15), the indirect protection by the morbidity-based strategy drops substantially, resulting in a higher proportion of elderly individuals infected than with the mortality-based strategy. A similar reversal occurs for infants. The mixed strategy—a combination of the morbidity-based and mortality-based strategies—is never the optimal strategy (Figure 3B), yet may be an advisable bet-hedging strategy when there is great uncertainty about the transmissibility of the circulating strain.

Estimates of *R*_0_ for interpandemic flu range between 1.0 and 2.4 for the A (H2N2) and A (H3N2) strains of influenza ([31,32] and references therein). Since influenza vaccines have been used in the US since 1944, these estimates may be based on partially vaccinated populations. Conservatively assuming that the populations in question had somewhere between no coverage at all and 13% coverage according to the contact-based strategy, these values of *R*_0_ (1.0 < *R*_0_ < 2.4) correspond to 0.06 < *T* < 0.26 in our model (See Protocol S1.) This range straddles the critical cross-points in Figure 3B, leaving some ambiguity as to which strategy will be most effective. We note, however, that the higher the transmissibility, the more dire the public health situation, and mortality-based strategies are predicted to be more effective for highly contagious strains.

### Highly Virulent Influenza

The demographic-specific mortality rates reported for influenza vary considerably (Protocol S1 and Figure S3). To assess whether control recommendations can be generalized to new or anomalous strains of influenza, we analyzed a second, extreme scenario. Worldwide influenza pandemics are characterized by much higher levels of morbidity and mortality than annual epidemics, and have occurred three times in the last century. The 1918–1919 “Spanish Influenza” caused more than 500,000 deaths in the US and an estimated 20 million deaths worldwide [33]. Based on data from the 1918 pandemic, we modified our model in three respects: the number of people expected to seek medical attention upon infection, the age-specific mortality rates, and (consequentially) the age groups targeted by the mortality-based and mixed strategies.

Despite these substantial differences, the predictions for pandemic and interpandemic flu are qualitatively similar. The morbidity-based and contact-based strategies outperform mortality-based strategies in terms of resulting mortality for low values of *T,* but not for higher values. There is a quantitative difference, however, in that the transition point between these two regimes happens at a higher transmissibility for pandemic flu than for interpandemic flu (Figure 3D versus 3B). In other words, morbidity-based strategies are preferred for a wider spectrum of pandemic flu strains than of interpandemic flu strains. This stems, in part, from the much larger size of the high-risk population (adults) for pandemic flu. Under vaccine limitations (13% in this case), the mortality-based strategy protects a much smaller fraction of the pandemic high-risk population than of the interpandemic high-risk population. We have found that increasing the vaccination level to 20% does not change the qualitative results (shown in Protocol S1 and Figure S4). Patel et al. have recently performed a similar sensitivity analysis on vaccine availability [34].

The reproductive number (*R*_0_) for the 1918 Spanish Influenza is estimated to have been between 1.8 and 4.0 [29,35], corresponding to *T* between 0.09 and 0.43 in our model (see Protocol S1). Once again, this range straddles the critical cross-point in Figure 3D, leaving some ambiguity as to which strategy will be most effective. It can be seen, however, that mortality-based strategies are predicted to be more effective across the upper two-thirds of this interval.

### Multiple Introductions

**Figure 6:**
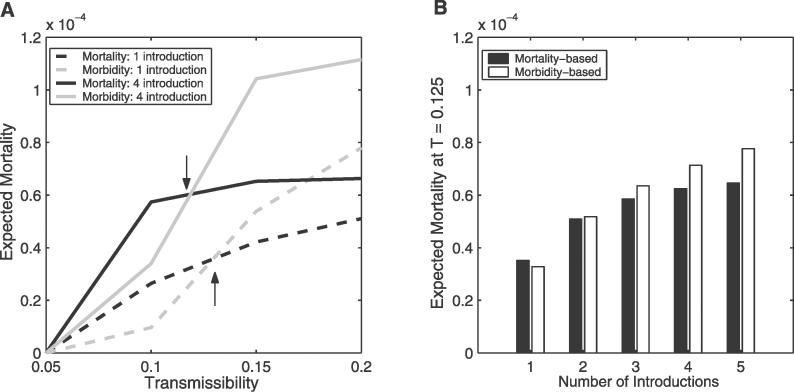
The Epidemiological Impact of Multiple Introductions of Disease (A) The morbidity-based strategy is more effective than the mortality-based strategy when *T* is less than 0.13 only if there is only a single introduction of disease. With four introductions of disease, however, the morbidity-based strategy becomes less effective (and is the preferred strategy only when *T* is less than 0.12.) (B) At *T* = 0.125, the morbidity-based strategy is superior to the mortality-based strategy when there is a single introduction, but inferior when there is more than one introduction.

Most communities do not exist in isolation, and thus experience multiple independent introductions of the virus during a typical flu season. Many models of vaccination strategies [8,9], however, ignore this possibility. To better understand the probability and rates of new importations of flu, one must consider a meta-population model that includes connectivity among cities. Here we address the consequences of multiple introductions, but not the likelihood of such events in the first place. For mathematical simplicity, we assumed that multiple independent introductions occur simultaneously (and initial cases are chosen randomly) at the start of an outbreak, which yields conservative estimates of their detrimental impact. The probability of an epidemic increases with the number of introductions for all strategies, thereby reducing the advantage of the morbidity-based and contact-based strategies for mildly transmissible strains. For example, if there are four independent introductions of flu, morbidity-based strategies are inferior to mortality-based strategies above *T* = 0.12 (*R*_0_ = 2.1). In contrast, this shift takes place at *T* = 0.13 (*R*_0_ = 2.3), when there is a single importation of disease (Figure 6).

### Delayed Intervention

**Figure 7:**
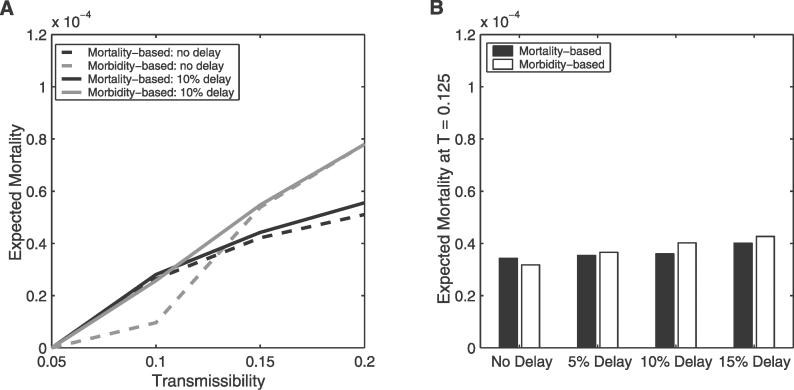
The Epidemiological Impact of Delayed Vaccination (A) The morbidity-based strategy is more effective than the mortality-based strategy when *T* is less than 0.13 only if there is there is no delay in vaccination. When vaccines are given after 10% of the population has already been infected, the morbidity-based strategy becomes relatively less effective (and is the preferred strategy only when *T* is less than 0.11). (B) At *T* = 0.125, the morbidity-based strategy is superior to the mortality-based strategy when there is no delay, but inferior for any amount of delay. Each of the values is an average taken across 500 epidemic simulations on the contact network.

A similar analysis provides insight into the impact of a delay in intervention until after an outbreak is already in progress, as occurred during the 2000–2001 flu season [36]. This scenario may also be particularly relevant to pandemic influenza, for which vaccines may only become available well into an outbreak, if at all. We simulate the implementation of vaccination after a certain proportion of the population has already been infected. We call this proportion “delay.” The morbidity-based strategies are more sensitive to such delays than mortality-based methods are (Figure 7). They are predicted to be inferior above *T* = 0.11 (*R*_0_ = 1.9) if there is a 10% delay in vaccination, compared to *T* = 0.13 (*R*_0_ = 2.3) when there is no delay.

Figures 6 and 7 suggest that a delay in vaccination may be less detrimental than multiple introductions of disease into a population. Multiple independent introductions of disease provide multiple independent opportunities to spark a large-scale epidemic. In the absence of vaccination, the probability of an epidemic increases considerably as the number of independent introductions increases (Protocol S1). In contrast, a delay in vaccination allows a single case to grow into a connected cluster of cases, which are not independent of each other with respect to the numbers and the identities of their contacts. The probability of an epidemic increases with the number of individuals in the initial cluster, but not as quickly as it does with the addition of independent cases.

### Conclusion

In this study, we have applied the analytical methods of contact network epidemiology to evaluate current and proposed influenza vaccination priorities. In contrast to prior studies [9,34], we have modeled a relatively large population and the entire spectrum of viral transmission rates possible for influenza; in addition, we have accounted for multiple introductions of disease and the possibility of a delay in vaccination. The efficacy of mortality-based strategies (like the CDC 2004 vaccination priorities [12]) and morbidity-based strategies (like school-based vaccination [8,9]) depend on (i) the transmissibility (reproductive number) of the strain, (ii) age-specific mortality rates, (iii) the vulnerability of the community to multiple introductions, and (iv) the timing of implementation. With respect to minimizing mortality, mortality-based strategies are generally preferred to morbidity-based strategies for strains with high transmission rates and in communities experiencing either delayed intervention or multiple introductions.

Thus, mortality-based strategies may be the prudent choice for outbreaks of new or atypical strains of influenza, when public health officials may not have reliable estimates for all (or any) of the first three inputs, and vaccination may be delayed. The predictions appear to hold for a range of age-specific mortality distributions estimated for past outbreaks of epidemic and pandemic flu. Although this suggests that similar recommendations may be appropriate for pandemic flu, they will be irrelevant in the very likely case that vaccines are not available at the start of an outbreak.

If more precise estimates of the key inputs become available, then this approach can be applied to design optimal (rather than just prudent) priorities. To reduce the existing uncertainty in estimates of influenza transmission and mortality rates, we must improve surveillance methods for gathering real-time data and develop new statistical methods for examining data from both historical and future outbreaks, as were developed to estimate *R*_0_ for SARS during the 2003 outbreaks [37]. Current estimates of flu transmission rates are based primarily on compartmental models of disease transmission ([31,32] and references therein). Some of the variation in the estimates of *R*_0_ may stem from variation in contact patterns among different populations rather than intrinsic variation in the probability of disease transmission between individuals who come in contact with one another. While compartmental models often do not capture such contact heterogeneity, contact network models allow one to factor out variation in contact patterns when estimating transmission rates. Thus, the development of better estimation methods using contact network models may yield more accurate estimates of some key epidemiological parameters.

## Supporting Information

Figure S1 (Normalized) Degree Distributions for Various Demographic Groups before and after Vaccination (with the Interpandemic Mortality-Based Strategy and the Morbidity-Based Strategy) (65 KB PDF)

Figure S2 Variation in the Size of Epidemic and Total Mortality Predicted for Mortality-Based and Morbidity-Based Strategies across 100 Networks with 100% Variation in Contact Parameters (64 KB PDF)

Figure S3 Epidemiological Predictions for Five Different Estimated Influenza Mortality Distributions (67 KB PDF)

Figure S4 Total Mortality at a 20% Vaccination Coverage Level (61 KB PDF)

Figure S5 Results from Simulation Demonstrate that the Two Methods of Modeling Vaccine Efficacy Give Similar Results (65 KB PDF)

Figure S6 Results for Total Mortality Rate with Variation in Infectivity and Susceptibility (58 KB PDF)

Protocol S1 Quantitative Comparison of Influenza Vaccination Programs (159 KB PDF)

## Abbreviations

CDC: United States Centers for Disease Control and Prevention
