# Supplementary material for: A Comparative Analysis of Influenza Vaccination Programs
Source: PLoS Med. 2006 Oct 3;3(10):e387. doi: 10.1371/journal.pmed.0030387 (PMC1584413; doi:10.1371/journal.pmed.0030387)
Supplement: Supplementary file 1 [file pmed.0030387.sd001.pdf]

Supporting Information:

Quantitative Comparison of Influenza Vaccination Programs

Shweta Bansal, Babak Pourbohloul, Lauren Ancel Meyers

SUPPLEMENTAL METHODS

Urban Contact Network Generation

For this study, we generate plausible contact networks for an urban setting using demographic information for the Greater Vancouver Regional District, which is the third largest metropolitan area in Canada. We use publicly available data from sources such as Statistics Canada to estimate the distribution of ages, household sizes, school and classroom sizes, hospital occupancy, workplaces, and public spaces [S1-S4]. Qualitatively similar age and household size distributions are found for other cities in Canada ranging in population sizes from 120,000 to 4.6 million [S5]. We begin assembling the urban network by choosing 100,000 households at random from the Vancouver household size distribution [S1], which yields approximately 257,000 people according to a mean household size of approximately 2.6. Based on ages assigned from the measured Vancouver age distribution [S2], each member of the population is assigned to activities: to schools according to school and class size distributions [S3]; to occupations according to (un)employment data; to hospitals as patients and caregivers according to hospital employment and bed data [S4]; to nursing homes according to nursing home occupancy data; and to other public places.

To model heterogeneities in contact patterns, we create random connections (edges) between individuals (nodes) based on the location and nature of their overlapping daily activities. Individuals in households are connected with probability 1, while individuals encountering others in public places are connected with probabilities ranging from 0.003 to 0.3. Each school

and hospital is subdivided into classrooms or wards. Pairs of students and pairs of patients within these subunits are connected with higher probability than pairs associated with different subunits. Teachers are assigned to classrooms and connected stochastically to appropriate students. Caregivers are assigned wards and then connected to appropriate patients. There are also low probability neighborhood contacts between individuals from different households.

Epidemiological Analysis

The methods described in this section are derived and described fully in Ref S6. Here, we only present the epidemiological equations with a few motivating details. Our contact network models are *semi-directed* – containing both undirected and directed edges. In a semi-directed network, each vertex (individual) has an *undirected-degree* representing the number of undirected edges joining the vertex to other vertices as well as both an *in-degree* and an *out-degree* representing the number of directed edges coming *from* other vertices and going *to* other vertices, respectively. The undirected-degree and in-degree indicate how many contacts can spread disease to the individual, and thus is related to the likelihood that an individual will become infected during an epidemic; and the undirected-degree and out-degree indicate how many contacts may be infected by that individual should he or she become infected, and thus is related to the likelihood that an individual will ignite an epidemic.

Given the degree distribution of the contact network within a population, one can analytically predict what will happen when an infectious disease like influenza enters the population. Let p_{jkm} be the probability that any given person in the population has in-degree equal to j, out-degree equal to k, and undirected-degree m. Let T be the transmissibility of the disease, that is, the average probability that transmission of the disease occurs between an

infected individual and a susceptible individual with whom they are in contact.

Network theory makes a technical distinction between outbreaks and epidemics. An outbreak is a causally connected cluster of cases which, by chance or because the transmission probability is low, dies out before spreading to the population at large. In an epidemic, on the other hand, the infection escapes the initial group of cases into the community at large and results in population-wide incidence of the disease. The crucial difference is that the size of an outbreak is determined by the spontaneous dying out of the infection, whereas the size of an epidemic is limited only by the size of the population through which it spreads.

To predict the fate of an outbreak, we use *probability generating functions*, to summarize useful information about network topology. Thus, if a graph has degree distribution p_{jkm}, then the probability generating function (PGF, henceforth) for p_{jkm} is

$$\Gamma(x, y, u) = \sum_{jkm} p_{jkm} x^j y^k u^m .$$

The average in-degree, out-degree, and undirected-degree are equal to: $\langle k_{in} \rangle = \sum_{jkm} jp_{jkm}$, $\langle k_{out} \rangle = \sum_{jkm} kp_{jkm}$, and $\langle k_{un} \rangle = \sum_{jkm} mp_{jkm}$. The average degrees can also be attained by evaluating the partial derivatives of $\Gamma(x, y, u)$ at $x = 1, y = 1$ and $u = 1$. We note that since every directed edge has an origin and a destination, the average in-degree equals the average out-degree $\left(\sum_{jkm} jp_{jkm} = \sum_{jkm} kp_{jkm} = \sum_{jkm} \frac{j+k}{2} p_{jkm} \right)$.

If you choose a random *directed* edge in the network and follow it to the nearest vertex, then the PGF for the number of the three types of edges (in, out, and undirected) emanating from that vertex other than the one that we arrived on is

$$H_d(x,y,u) = \frac{\sum_{jkm} jp_{jkm} x^{j-1} y^k u^m}{< k_{in} >}.$$

Likewise, if you choose a random *undirected* edge in the network and follow it to the nearest vertex, then the PGF for the various edges at that vertex is given by

$$H_u(x,y,u) = \frac{\sum_{jkm} mp_{jkm} x^j y^k u^{m-1}}{< k_{un} >}.$$

Using these methods, we can derive the reproductive ratio, R_0, the average size of an outbreak, $\langle s \rangle$, the size of an epidemic, S_e, the probability of an epidemic, P_e, and the probability that an individual with a certain (in- and undirected-) degree will become infected, v_{jm}.

The basic reproductive ratio: When calculating the expected number of new cases arising from an infection in a naïve population we consider the source vertex of the infection. That is, the initial case may arise through infection along a directed or undirected edge. Thus, if we know the source of the infection we can more accurately predict the R_0. In particular,

$R_0^d = T \frac{\sum_{jkm} j(k+m) p_{jkm}}{< k_{in} >}$ and $R_0^u = T \frac{\sum_{jkm} m(k+m-1) p_{jkm}}{< k_{un} >}$ respectively, where T is the average disease transmissibility and the second term is the average out-degree plus the average undirected-degree of a vertex that has become infected along a randomly selected edge. When we do not know anything about the transmission event that led to the initial infection, then our best estimate is

$$R_0 = T \frac{\sum_{jkm} (j(k+m) + m(k+m-1)) p_{jkm}}{< k_{in} > + < k_{un} >}.$$

Since R_0 is a product of both transmissibility (T) and the connectivity of the population, for a given value of T, different populations (networks) may have different values of R_0. If we are given range of R_0 values for a certain population, $p < R_0 < q$ for example, we can derive the lower and upper bounds for transmissibility that correspond to that range of R_0 as follows. Assuming we have no further information about the vaccination status of the population, we take the value of T that yields $R_0 = p$ for the population (network) with no vaccination (a worse-case scenario) and we take the value of T that yields $R_0 = q$ for the population (network) with maximum vaccination coverage (a best-case scenario.) For interpandemic flu, R_0 has been estimated to be $1 < R_0 < 2.4$. For an unvaccinated population, $R_0 = 1$ corresponds to $T = 0.06$ and for a population with maximum vaccination coverage (13%), $R_0 = 2.4$ corresponds to $T = 0.26$. Thus we estimate the transmissibility of interpandemic influenza to be $0.06 < T < 0.26$.

The average size of small outbreaks and the epidemic threshold: By nesting PGFs for the number of new infections emanating from an infected vertex one can construct a PGF for the size of a small outbreak, and hence derive the average size of a small outbreak:

$$\langle s \rangle = 1 + \frac{Tf_1^{\,j}\left[1 - T\left(f_m^{\,m(m-1)} - f_j^{\,jm}\right)\right] + Tf_1^{\,m}\left[1 - T\left(f_j^{\,jk} - f_m^{\,km}\right)\right]}{\left(1 - Tf_j^{\,jk}\right)\left(1 - Tf_m^{\,m(m-1)}\right) - T^2 f_j^{\,jm} f_m^{\,km}}$$

where $f_b^{\,a} = \dfrac{\sum_{jkm} a p_{jkm}}{\sum_{jkm} b p_{jkm}}$. When T is small, the average size of a small outbreak is finite, but $\langle s \rangle$ grows with increasing transmissibility, until it diverges when the denominator of the expression above reaches its first zero. This point marks the phase transition at which the typical outbreak ceases to be confined to a finite number of cases and expands to a large-scale epidemic covering most of the network. This transition happens when T is equal to

the critical transmissibility T_c, given by

$$T_c = \frac{\left(f_j^{jk} + f_m^{m(m-1)}\right) \pm \sqrt{\left(f_j^{jk} + f_m^{m(m-1)}\right)^2 - 4\left(f_j^{jk} f_m^{m(m-1)} - f_j^{jm} f_m^{km}\right)}}{2\left(f_j^{jk} f_m^{m(m-1)} - f_j^{jm} f_m^{km}\right)}.$$

The expected size of a full-blown epidemic S_e: We can compute the size of the epidemic (the proportion of the population infected), S_e, for the case when T is larger than T_c. We first calculate the likelihood that infection of a randomly chosen individual will spark only a limited outbreak instead of a full-blown epidemic, and then take one minus that probability:

$$S_e = 1 - \sum_{jkm} p_{jkm} (1 + (a-1)T)^j (1 - (b-1)T)^m,$$

where a and b are the solutions to the self-consistent equations

$$a = \frac{\sum_{jkm} k p_{jkm} (1 + (a-1)T)^j (1 + (b-1)T)^m}{< k_{out} >} \text{ and } b = \frac{\sum_{jkm} m p_{jkm} (1 + (a-1)T)^j (1 + (b-1)T)^{m-1}}{< k_{un} >}.$$ We use numerical root finding methods (such as Newton's method) to solve for a and b.

The probability of a full-blown epidemic P_e: The expression for P_e comes from first calculating the likelihood that a single infection will lead to only a small outbreak instead of a full-blown epidemic, and then and then taking one minus the probability:

$$P_e = 1 - \sum_{jkm} p_{jkm} (1 + (\alpha-1)T)^k (1 - (\beta-1)T)^m,$$

where α and β are the solutions to the self-consistent equations

$$\alpha = \frac{\sum_{jkm} j p_{jkm} (1 + (\alpha-1)T)^k (1 + (\beta-1)T)^m}{< k_{in} >} \text{ and } \beta = \frac{\sum_{jkm} m p_{jkm} (1 + (\alpha-1)T)^k (1 + (\beta-1)T)^{m-1}}{< k_{un} >}.$$ We use numerical root finding methods (such as Newton's method) to solve for α and β.

The probability that an individual will be infected during an epidemic v_{jm} *:* The likelihood that an individual of in-degree *j* and undirected-degree *m* will be infected during an epidemic is equal to one minus the probability that none of his or her $j + m$ contacts will transmit the disease to him or her. The average probability that an individual at the (origin) end of a randomly selected directed edge is spared by an epidemic is *a*. For an individual at the end of a randomly selected undirected edge, this probability is *b*. Thus, the probability that one is not infected by a neighbor is the probability that the neighbor is infected but does not transmit disease *(1-a)(1-T)* for directed edges and *(1-b)(1-T)* for undirected edges, plus the probability that the neighbor is not infected, *a* for directed edges or *b* for undirected edges. These probabilities sum to *(1-T+Ta)* and *(1-T+Tb)* for directed and undirected edges, respectively. Thus, a randomly chosen vertex of in-degree *j* and undirected-degree *m* will become infected with probability

$$v_{jm} = 1 - (1 - T + Ta)^{j} (1 - T + Tb)^{m} .$$

Demographic-Specific Attack Rates: We calculate demographic-specific epidemiological risks by combining demographic information (age, occupation, etc.) for each member of the population with the v_{jm}, defined above. We first divide the population into 14 demographic groups:

Demographic Group (g)	***Demographic Group Description***
1	Infants (age < 3)
2	Toddlers (3 ≤ age < 5)
3	Children (5 ≤ age < 18)
4	Adults (18 ≤ age < 50)
5	Elderly (age > 50)
6	Nursing home residents
7	Infants in daycare
8	Toddlers in preschool
9	Health care workers
10	Nursing home workers
11	Day care workers
12	Preschool workers
13	Teachers (and school staff)
14	Unemployed

For each demographic group (*g*), we find the expected number of infections (N_g) at a particular transmission probability *T* by summing the probabilities of infection (v_{jm}) across all individuals in that group. We denote the in-degree and undirected degree of an individual (*i*) by $j(i)$ and $m(i)$, respectively:

$$N_g = \sum_{i \in g} v_{j(i)m(i)} \qquad \forall g \in [1,14].$$

Age-Specific Mortality: The predicted number of deaths in the population caused by an epidemic (*M*) is the product of the predicted number of infections in each of the age groups (demographic groups 1 through 5 in the Table above) and the age-specific mortality rate (R_g) specified in Table 3:

$$M = \sum_{g=1}^{5} N_g * R_g$$

The predicted total mortality rate for the population is M normalized by the population size.

Multiple Introductions: We can also analytically predict the probability of an epidemic given independent multiple introductions of disease into a population. For a given number of introductions, n, the probability of an epidemic is given by:

$$\boldsymbol{\pi}_n = 1 - (1 - P_e)^n,$$

where, P_e, is the probability of an epidemic assuming a single introduction. We note that the calculation of $\boldsymbol{\pi}_n$ above assumes that all n introductions occur independently at the outset of an outbreak. This assumption yields an upper bound estimate for the probability of an epidemic with multiple introductions.

Epidemic Simulation

We verify the analytic predictions using simulations of a Susceptible-Infectious-Recovered (SIR) model. The simulations are initialized with an entirely susceptible population, except for a single infected case (*patient zero*). An infected vertex passes the disease on to each of its neighbors (those with whom that individual has disease-causing contacts) with probability T (the average transmission probability). This process continues until the population no longer includes any susceptible individuals that are in contact with any infected individuals. Once an individual has had the chance to infect its neighbors, it is immediately moved into the recovered class.

SUPPLEMENTAL ANALYSIS

Network Properties of Demographic Groups

Here we describe basic properties of the simulated urban networks that we have analyzed. The epidemiological calculations consider the degree distribution of the network (as described in the previous section.) Recall that most of the edges in our network are undirected and many individuals have the same out-degree as in-degree, with the exception of health care workers and individuals who are at high risk for complications due to flu. In Figure S1, we show the in-degree distributions for the total population and select demographic groups before and after vaccination by the morbidity and mortality-based strategies. Children have a much higher mean in-degree (24.1) than adults and elders (10.7 and 10.6, respectively). Figure S1c illustrates that the contact patterns for adults are relatively unaffected by both the morbidity- and mortality-based strategies. The morbidity-based strategy primarily alters the degree distribution of children (Figure S1b) and the mortality-based strategy primarily alters the degree distribution of elders (Figure S1d). The mortality-based strategy does not affect the degree distribution of the total population a great deal (Figure S1a) as it effectively only targets small groups, either due to low vaccine efficacy levels (elders) or few individuals in the demographic group (infants.)

Sensitivity to Population Structure

The urban networks are stochastically generated, yielding Poisson distributions of contact numbers within each setting (schools, hospitals, workplaces, etc.). To achieve this, we specify setting-specific probabilities that determine whether or not any given pair of individuals in the same location will have an edge drawn between them. We examined the sensitivity of our results to the specific probabilities used in generating the network. First, we generate 100 networks

each with 5000 households. (The smaller population size allowed for more extensive sensitivity analyses. In prior studies, we found that epidemiological predictions for small urban networks apply to large urban networks [S5], and thus we expect that these sensitivity results will also apply to large networks.) To generate variation in these networks, we draw contact probabilities between individuals from a Gaussian distribution, and allow them to deviate by up to 100% from the original contact parameters (which range from 0.003 to 0.3 depending on the location/nature of the interaction). The stochastic formation of edges according to these probabilities yields 100 unique networks, each with its own degree distribution. We then vaccinate each population according to the morbidity-based and mortality-based strategies, as described in the methods section. The dashed lines in Figure S2 indicate the standard deviations for each epidemiological prediction (morbidity and mortality) across the 100 networks. The small variation in the predictions indicates that our results are robust to stochastic variation in network structure. Even with a 100% deviation in contact structure, the morbidity and mortality-based strategies are superior for lower and higher values of T, respectively. The value of T at which preferred strategy switches falls in the range [0.10, 0.14]. In the main text, we report a transition point of T=0.11. These results suggest that even a 100% uncertainty in contact rates produces an uncertainty of [-0.01, 0.03] around the transition point.

Sensitivity to Mortality Rates

To evaluate the sensitivity of our predictions to variations in virulence among different strains of influenza, we evaluated vaccination strategies for two markedly different estimated mortality distributions. Here we extend this analysis to several other estimated influenza mortality distributions. We compare the total mortality caused by an influenza epidemic after the

population has been vaccinated with a morbidity-based or mortality-based strategy for five different age-specific mortality distributions. The first two mortality distributions are the focus of the main text. The third is a different estimate for 1918 flu mortality rates that includes high mortality rates for adults and the elderly; and the remaining two are U-shaped mortality distributions reported for the epidemics of 1892 and 1936 [S8]. The mortality-based strategy is designed to target the demographic groups with the highest mortality rates, and thus varies from one mortality distribution to the next. The morbidity-based strategy is identical across all five mortality distributions, targeting school children and staff as specified in the main text. We are particularly interested the cross-points (in transmissibility values) where the mortality-based strategy becomes superior to the morbidity-based strategy. These lie between T = 0.15 and T = 0.20 for the three additional mortality distributions shown in Figure S3, very close to that predicted for the 1918 mortality distribution considered in the main text, further suggesting that the results are fairly insensitive to uncertainties in estimates of influenza mortality rates.

Sensitivity to Vaccine Coverage Level

We test the sensitivity of our results to a change in the vaccine coverage level. The vaccine priorities in the main text are implemented at a 13% coverage level. Here, we implement the morbidity-based strategy (school children and staff) and mortality-based strategy (based on the second mortality distribution of Figure S3) at a 20% coverage level. (During the influenza vaccine shortage of 2004, enough vaccine was available to cover 20% of the population.) Figure S4 shows that the increase in vaccination coverage produces a smaller mortality rate for both strategies but the comparison is qualitatively similar: the mortality-based strategy outperforms the morbidity-based strategy for higher values of transmissibility.

Vaccine Effectiveness and Efficacy

Vaccine efficacy is defined as $E = 1 -$ (attack rate among vaccinated population/attack rate among unvaccinated population). To model vaccination of a proportion C of a demographic group at an efficacy of E, we remove a fraction $C*E$ of individuals in the group from the network entirely. This reduces the attack rate in the vaccinated group by exactly a fraction E, which yields an efficacy of E. Although this method technically assumes that the vaccine has 100% effectiveness for the fraction E of the vaccinated group and no effectiveness on the remaining fraction 1-E of vaccinated individuals, it provides a reasonable model for the more realistic scenario in which most vaccinate individuals enjoy some level of protection. To test that the 100% effectiveness model is a reasonable approximation, we have compared its predictions to simulations in which all vaccinated individuals have partially reduced susceptibility. In particular, to vaccinate a fraction C of a group with a vaccine of efficacy $E > 0$, we select C individuals at random from the group and reduce the transmission probability along all edges leading to each of those individuals by a factor $(1-E^{1/m}) / T(1-b)$, where m is the undirected degree of the node, T is the average transmissibility, and b is the average probability that an individual at the end of a randomly selected undirected edge is spared by an epidemic. This factor is based on the fact that the probability of infection for each vaccinated individual will be $1-E$, and this yields a reduction in the attack rate in the group of E. We then simulate the spread of disease through the network. We find that mathematical predictions assuming 100% effectiveness closely match the results of these simulations, as illustrated in Figure S5.

Sensitivity to Variation in Infectivity and Susceptibility

There is certainly heterogeneity in influenza infectivity and susceptibility among individuals. Some of the heterogeneity is caused by variation in contact patterns [S9]. Individuals with more contacts will have greater opportunities to catch and spread disease. Our models explicitly capture this source of variation. The remaining heterogeneity in transmission rates is caused by intrinsic physiological and behavioral differences among individuals. Our analytical calculations allow for such heterogeneity so long as it is distributed somewhat randomly with respect to the structure of the population. That is, there should not be significant correlations between individual contact patterns and individual likelihoods of infection and/or transmission. There is evidence, however, that such correlations may exist. Cauchemez et al. statistically argue that children have a higher infectiousness and a higher susceptibility than adults *per contact* [S10].

We have modified our contact network to explicitly model this diversity in transmissibility. Cauchemez et al. suggest that, within a household, susceptible children (under 15 years of age) have a 15% higher susceptibility per day of contact with an infectious household member compared to susceptible adults. They estimate that infectious children have 84% higher infectivity per day of contact with susceptible household members compared to infectious adults (0.26 and 0.48 for adults and children, respectively). Finally, they find that the expected infectious periods of flu for children and adults are 3.6 and 3.9 days, respectively [S10].

To test the sensitivity of our model to such heterogeneity, we make the extreme assumption that the different transmission probabilities will also hold for contacts outside the home. Accordingly, contacts between children were given the highest probability of transmission (T_{CC}), followed by those from children to adults (T_{CA}), from adults to children (T_{AC}), and finally between adults (T_{AA}). We assigned probabilities of transmission to the edges in the network based on the ratios between these values calculated from the data reported in [S10]. In particular,

each transmissibility is the average probability of transmission between an infectious and susceptible individual during the infectious period, or $1-(1-p)^{\tau}$ where τ is the length of the infectious period and *p* is the per day probability of infection. Borrowing notation from [S10], for an edge pointing from an infected individual of type *I* (child or adult) to a susceptible individual of type *J* (child or adult) this is equal to $1-\left(1-\beta_I \varepsilon_J\right)^{\tau_I}$ where β_I is the per day probability of transmission from *I* to one of his or her contacts, τ_I is the duration of *I*'s infectious period, and ε_J is the susceptibility factor of individual *J*. The table below gives the ratios that we used to determine transmissibilities in the model.

**Contact Type (I → J)**	**Transmissibility calculated from estimates reported [S10]** $1-\left(1-\beta_I \varepsilon_J\right)^{\tau_I}$	**Ratio of T_{IJ} to T_{AA}**
Adult → *Adult*	$1-(1-0.26)^{3.9}$	1
Adult → *Child*	$1-(1-0.26 \cdot 1.15)^{3.9}$	1.09
Child → *Adult*	$1-(1-0.48)^{3.6}$	1.31
Child → *Child*	$1-(1-0.48 \cdot 1.15)^{3.6}$	1.37

For a range of possible values for T_{AA}, we assigned transmissibilities according to these ratios and ran 250 SIR simulations on the network for each of three scenarios: no vaccination, mortality-based vaccination, and morbidity-based vaccination. We also calculated the average transmissibility *T* across all contacts in the network, with which we made analytical predictions. In Figure S6, we compare analytical predictions that consider only the average transmissibility *T* (lines) to the outcome of these simulations (circles). Our current analytic methods give qualitatively similar results to those of the simulations. Although there is a bit of a discrepancy

between the predicted and simulated results for the morbidity-based strategy and consequently for the transition point between the two strategies, the important results still hold. That is, the mortality-based strategy remains advisable over a large range of highly contagious strains (even larger in the simulations than the analytics), and the cross-point between the two strategies remains within the range of estimates of R_0 for interpandemic flu.

Although the results in Figure S6 may seem counter-intuitive, they point to some of the important features of our model. Generally, the morbidity-based strategy reduces mortality via herd immunity while the mortality-based strategy reduces mortality by directly protecting those with highest mortality rates. By assuming that edges to and from children have higher transmissibilities, it becomes more difficult to achieve herd immunity via the morbidity-based strategy. Despite the fact that the morbidity-based strategy targets a core group (50% of all children), it leaves a substantial population of children with high degree and high infectiousness which continues to act as a core group sufficient enough to reach the high-risk individuals. The mortality-based strategy is relatively unaffected by the heterogeneity because it continues to protect the same fraction of high-risk individuals (in neither case does it achieve much herd immunity).

This sensitivity analysis was based on a fairly extreme form of heterogeneity in transmission probabilities. In reality, variation in transmission probabilities outside households may be less demographically-structured, in which case, the assumption that variation in transmission probabilities is fairly random with respect to network structure may be valid. Similar analytic methods that explicitly capture demographic-specific patterns of transmission rates give more exact predictions for this extreme scenario, but are beyond the scope of this paper.

References

S1. Household Size, census metropolitan areas. Statistics Canada (2001)

S2. 2001 Census Profile of British Columbia's Regions: Greater Vancouver Regional District, *BC Stats* (2003)

S3. Vancouver School Board, December 2002 Ready Reference (2002).

S4. The British Columbia Health Atlas, Centre for Health Services and Policy Research (2002)

S5. Pourbohloul B, Meyers LA, Skowronski DM, Krajden M, Patrick DM, et al. (2005) Modeling Control Strategies of Respiratory Pathogens. Emerg Infect Dis. 11: 1249-1256

S6. Meyers LA, Newman MEJ, Pourbohloul B (2006) Predicting epidemics on directed contact networks. JTB. In press.

S7. Newman MEJ (2002) Spread of epidemic disease on network. Phys. Rev. E 66, 016128

S8. Dauer CC, Serfling RE (1961) Mortality from Influenza, 1957-1958 and 1959-1960. Am Rev Respir Dis. 83 (2 Suppl): 15-26.

S9. Addy CL, Longini IM, Harber M (1991) A Generalized Stochastic Model for the Analysis of Infectious Disease Final Size Data. Biometrics. 47: 961-974.

S10. Cauchemez S, Carrat F, Viboud C, Valleron AJ, Boelle PY (2004) A Bayesian MCMC approach to study transmission of influenza: application to household longitudinal data. Stat Med. 23: 3469-3487.

S11. Thompson WW, Shay DK, Weintraub E, Brammer L, Cox N et al. (2003) Mortality associated with influenza and respiratory syncytial virus in the United States. JAMA. 289: 179–86.

S12. Simonsen L, Clarke MJ, Schonberger LB, Arden NH, Cox NJ et al. (1998) Pandemic versus epidemic influenza mortality: a pattern of changing age distribution. J Infect Dis. 178: 53–60.
