# Supplementary material for: A Comparative Analysis of Influenza Vaccination Programs
Source: PLoS Med. 2006 Oct 3;3(10):e387. doi: 10.1371/journal.pmed.0030387 (PMC1584413; doi:10.1371/journal.pmed.0030387)
Supplement: Supplementary file 2 [file pmed.0030387.sg001.pdf]

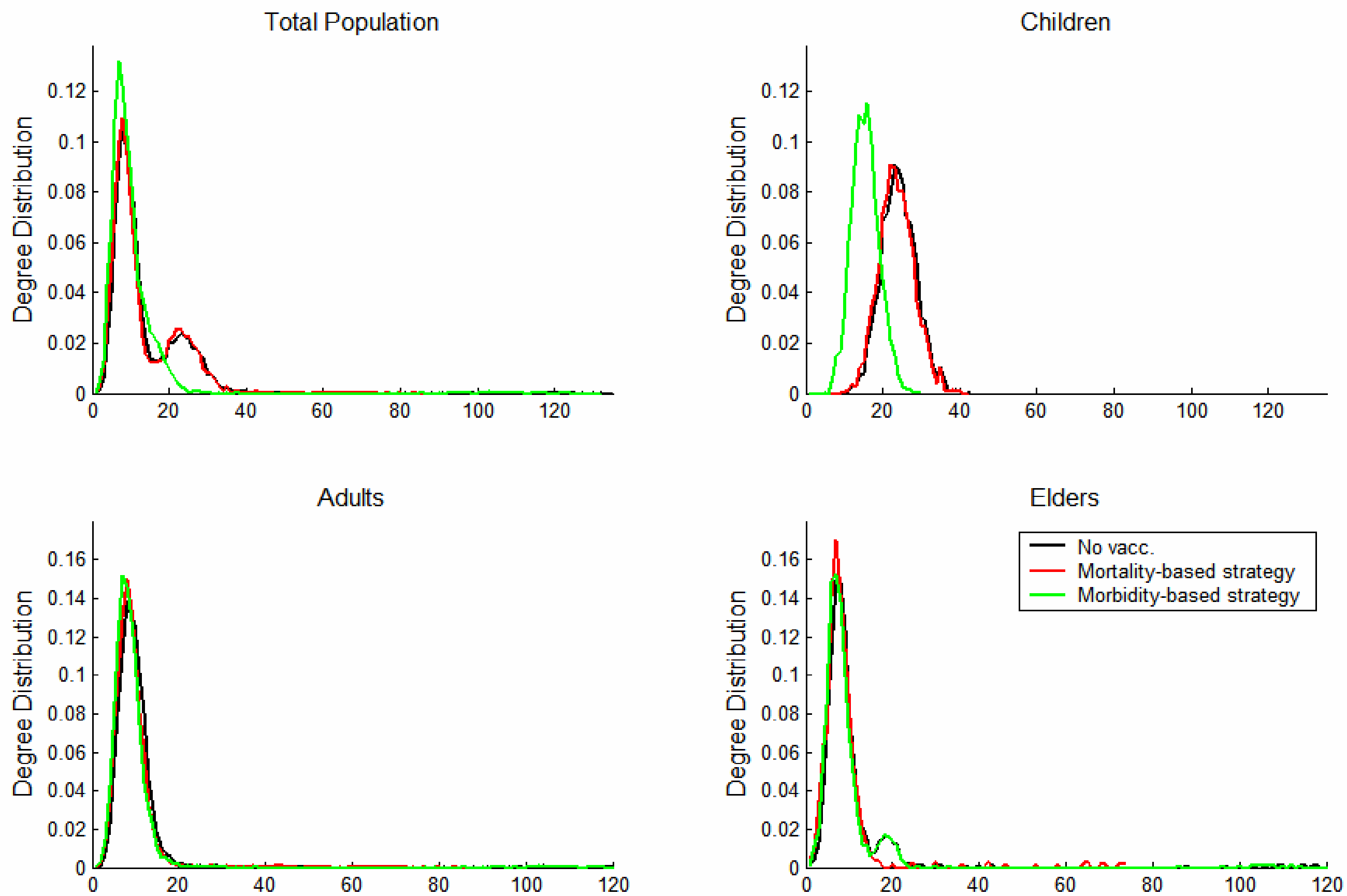

Figure S1: (Normalized) Degree distributions for various demographic groups before and after vaccination (with the interpandemic mortality-based strategy and the morbidity-based strategy.) Vaccinated individuals are not included in the distributions shown for the two strategies.
