# Supplementary material for: A Comparative Analysis of Influenza Vaccination Programs
Source: PLoS Med. 2006 Oct 3;3(10):e387. doi: 10.1371/journal.pmed.0030387 (PMC1584413; doi:10.1371/journal.pmed.0030387)
Supplement: Supplementary file 3 [file pmed.0030387.sg002.pdf]

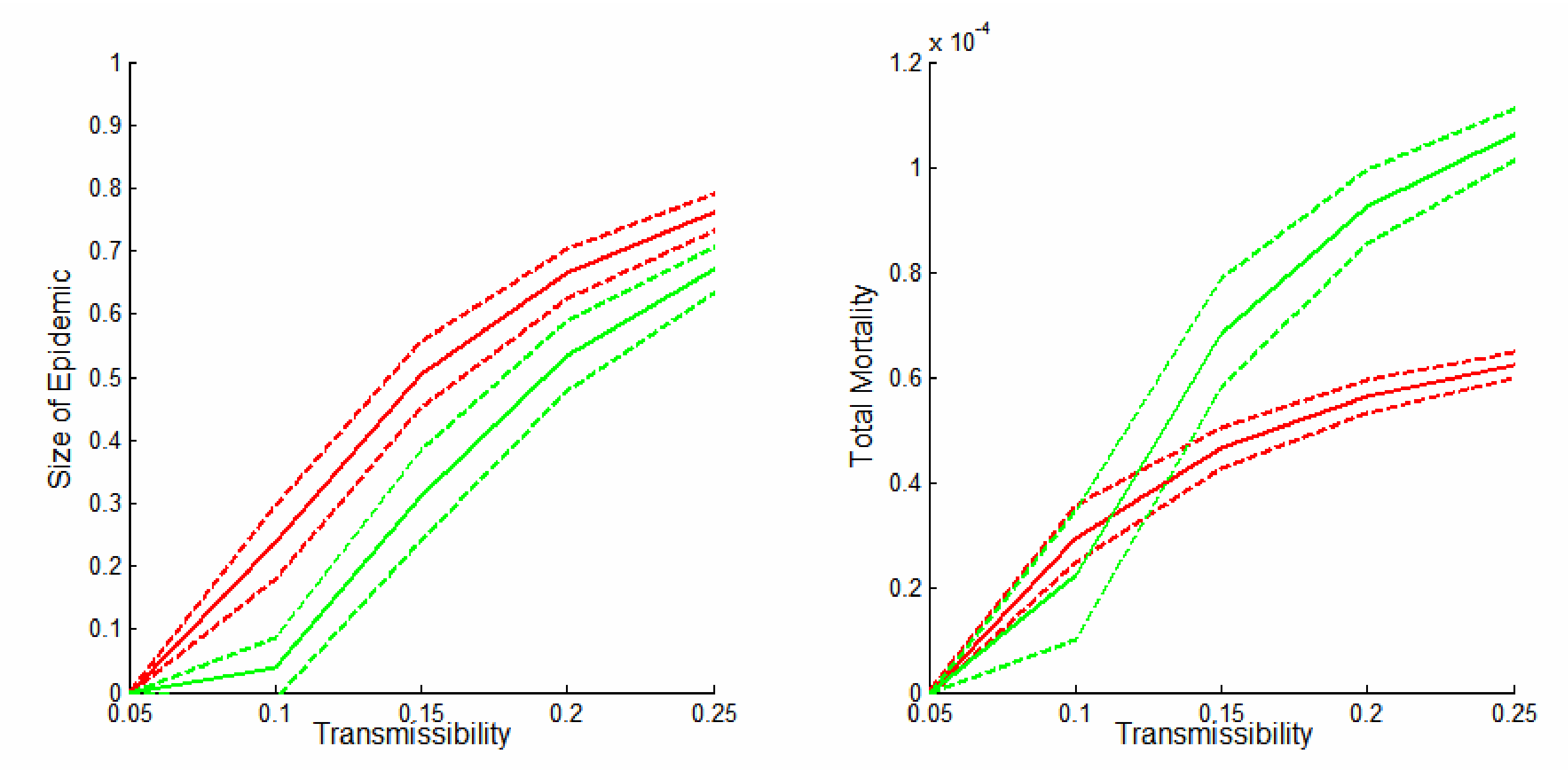

Figure S2: Variation in the size of epidemic and total mortality predicted for mortality-based (red) and morbidity-based (green) strategies across 100 networks with 100% variation in contact parameters. Dashed lines indicate standard deviations.
