# Supplementary material for: A Comparative Analysis of Influenza Vaccination Programs
Source: PLoS Med. 2006 Oct 3;3(10):e387. doi: 10.1371/journal.pmed.0030387 (PMC1584413; doi:10.1371/journal.pmed.0030387)
Supplement: Supplementary file 4 [file pmed.0030387.sg003.pdf]

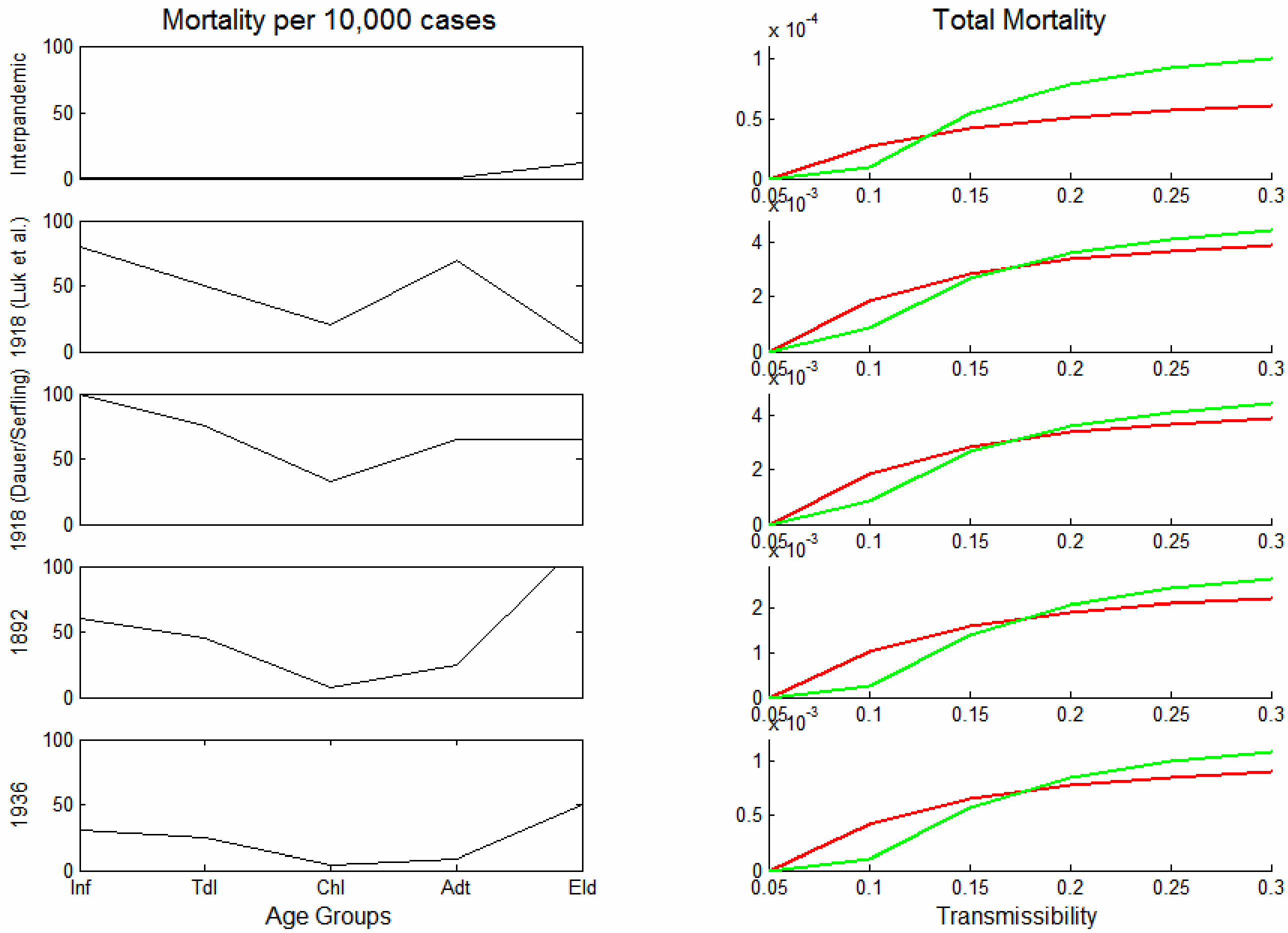

Figure S3: Epidemiological predictions for five different estimated influenza mortality distributions. Left: Mortality rates estimated for various influenza epidemics and pandemics [S8, S11, S12]. The top two distributions are considered in the main text. Right: Total mortality predicted for the mortality-based strategy (red) and morbidity-based strategy (green) assuming the corresponding distributions of mortality rates.
