# Supplementary material for: A Comparative Analysis of Influenza Vaccination Programs
Source: PLoS Med. 2006 Oct 3;3(10):e387. doi: 10.1371/journal.pmed.0030387 (PMC1584413; doi:10.1371/journal.pmed.0030387)
Supplement: Supplementary file 5 [file pmed.0030387.sg004.pdf]

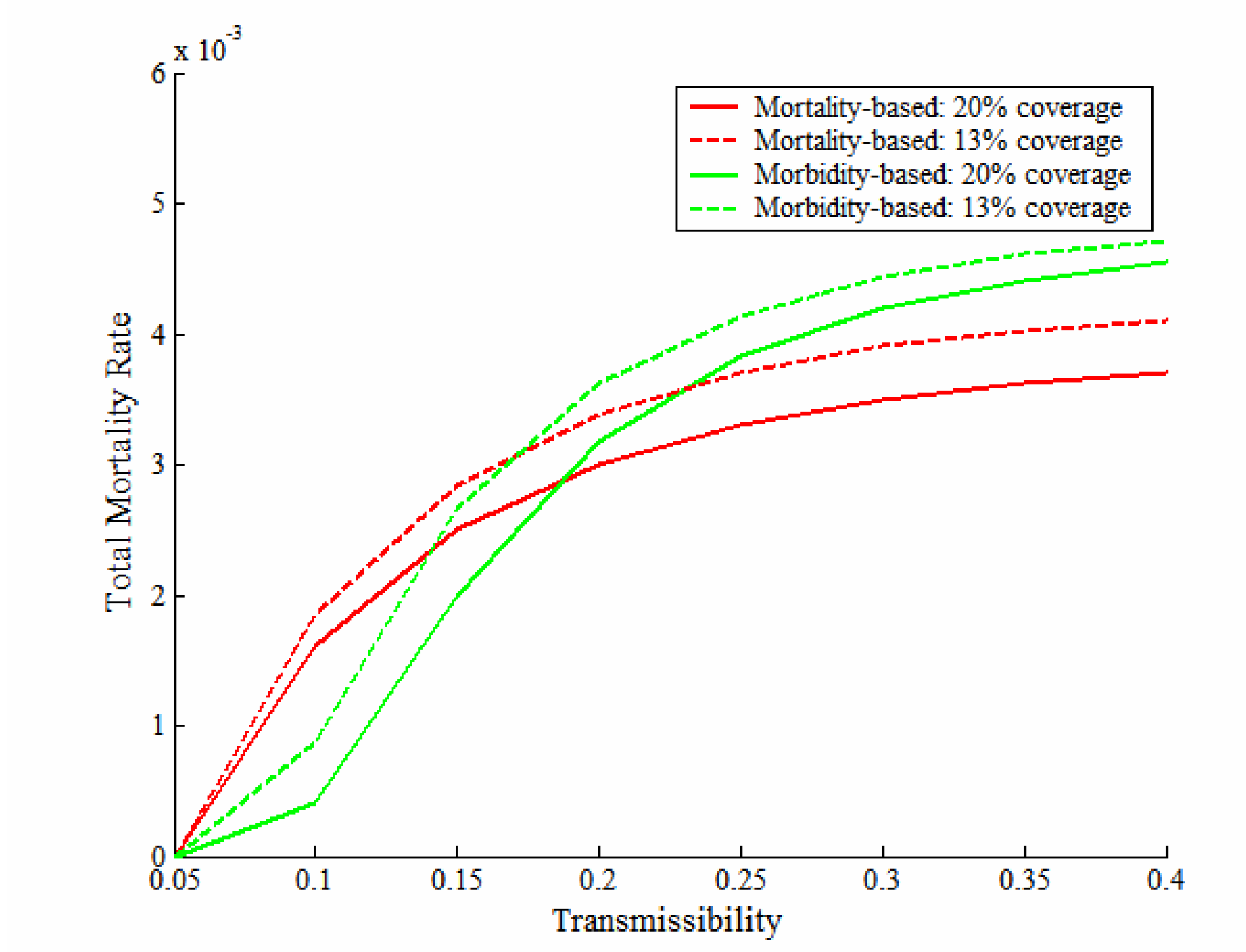

Figure S4: The total mortality at a 20% vaccination coverage level. The total mortality is lower for both strategies as compared to vaccination at a 13% coverage level (dashed lines). However, there still exists a point after which the mortality-based strategy is superior to the morbidity-based.
