# Supplementary material for: A Comparative Analysis of Influenza Vaccination Programs
Source: PLoS Med. 2006 Oct 3;3(10):e387. doi: 10.1371/journal.pmed.0030387 (PMC1584413; doi:10.1371/journal.pmed.0030387)
Supplement: Supplementary file 6 [file pmed.0030387.sg005.pdf]

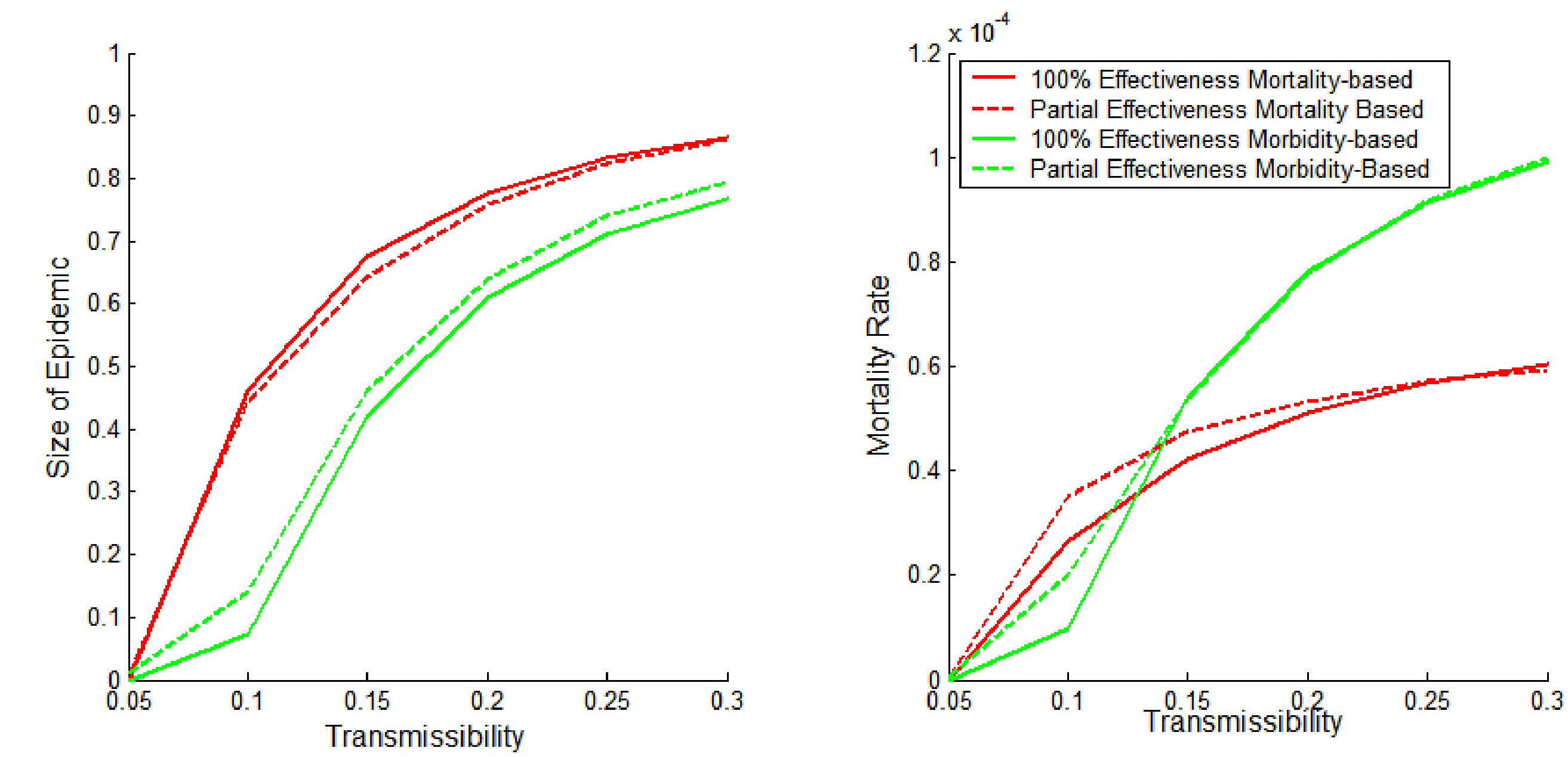

Figure S5: Results from simulation demonstrate that the two methods of modeling vaccine efficacy give similar results. Results are shown for the mortality-based and morbidity-based strategies. 'Partial effectiveness' refers to the vaccination of a proportion *C* of the population with effectiveness *E*. '100% effectiveness' refers to the vaccination of a proportion *C**E* of the population with effectiveness 1.
