# Supplementary material for: A Comparative Analysis of Influenza Vaccination Programs
Source: PLoS Med. 2006 Oct 3;3(10):e387. doi: 10.1371/journal.pmed.0030387 (PMC1584413; doi:10.1371/journal.pmed.0030387)
Supplement: Supplementary file 7 [file pmed.0030387.sg006.pdf]

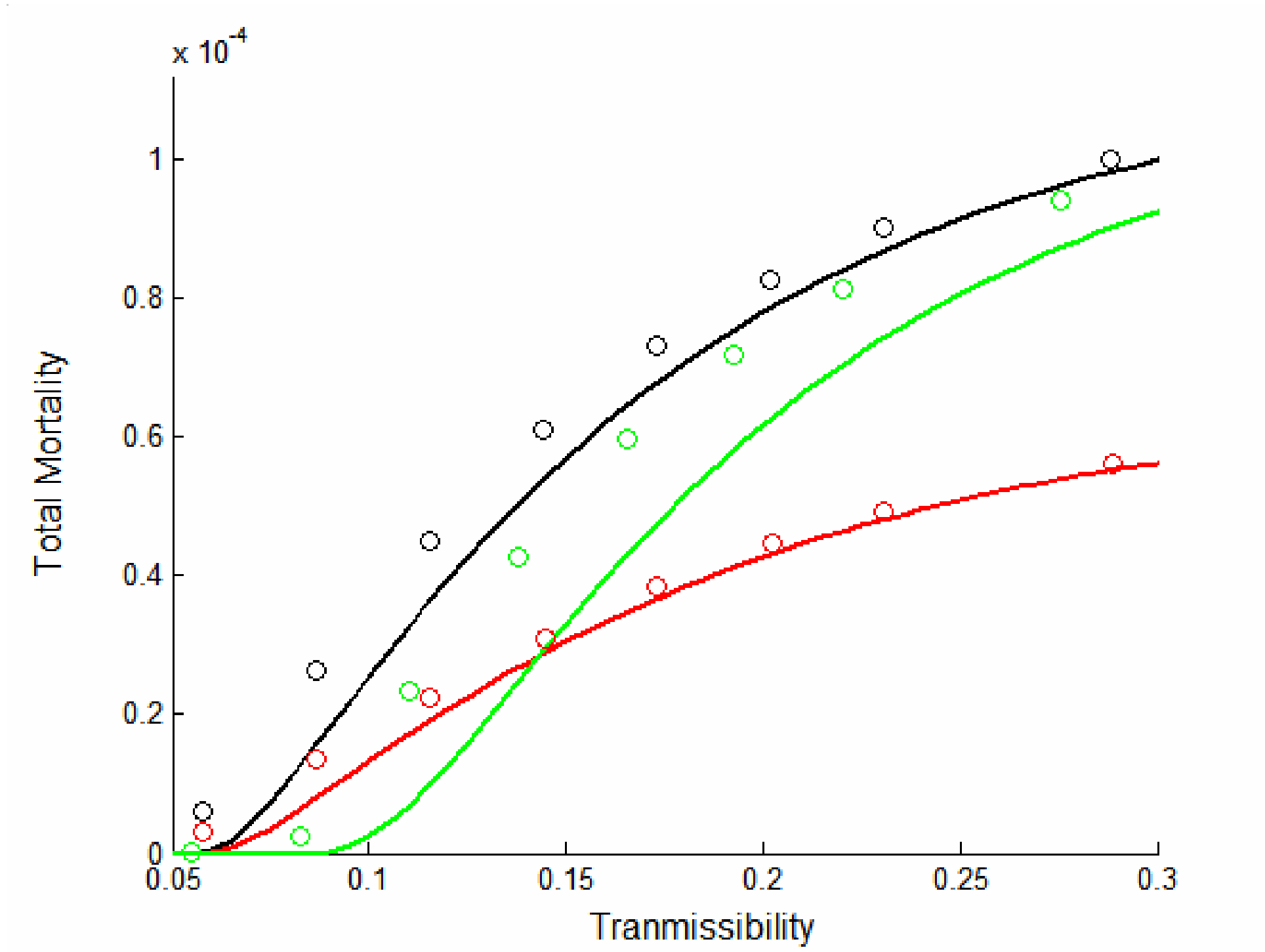

Figure S6: Results for total mortality rate with variation in infectivity and susceptibility. The x-axis corresponds to the average transmissibility across all edges in the network. Circles are simulation results with individual variation in infectivity and susceptibility; lines are analytical results for the resulting average transmissibility on the same networks. The analytical calculations do not explicitly consider variation in infectivity and susceptibility on each edge.
